# Development of a prediction model for predicting the prevalence of nonalcoholic fatty liver disease in Chinese nurses: the first-year follow data of a web-based ambispective cohort study

**DOI:** 10.1186/s12876-024-03121-1

**Published:** 2024-02-14

**Authors:** Ying Che, Rongsong Tang, Heli Zhang, Min Yang, Rongmei Geng, Lin Zhuo, Peng Wang, Xianjing Hu, Yujie Zhou, Panfeng Wang, Siyan Zhan, Baohua Li

**Affiliations:** 1 Medical Examination Center, Peking university third hospital, Huayuan North Road No.49, Haidian District, Beijing, China; 2 Nursing Department, Peking University Third Hospital, Huayuan North Road No.49, Haidian District, Beijing, China; 3 Department of Rehabilitation Medicine, Peking University Third Hospital, Huayuan North Road No.49, Haidian District, Beijing, China; 4 Center for Clinical Epidemiology Research, Peking University Third Hospital, Huayuan North Road No.49, Haidian District, Beijing, China; 5 School of Nursing, Peking university, Xueyuan Road No. 38, Haidian District, Beijing, China; 6 General surgery department, Peking university third hospital, Huayuan North Road No.49, Haidian District, Beijing, China; 7 Oncology Radiotherapy Department, Peking university third hospital, Huayuan North Road No.49, Haidian District, Beijing, China

**Keywords:** Female, Nurses, Non-alcoholic fatty Liver Disease, Nomograms, Cohort studies

## Abstract

**Background:**

Nonalcoholic fatty liver disease (NAFLD) is gradually becoming a huge threat to public health. With complex working characteristics, female nurses had been found with high risk of NAFLD. To develop and validate a prediction model to predict the prevalence of NAFLD based on demographic characteristics, work situation, daily lifestyle and laboratory tests in female nurses.

**Methods:**

This study was a part of the Chinese Nurse Cohort Study (The National Nurse Health Study, NNHS), and data were extracted from the first-year follow data collected from 1st June to 1st September 2021 by questionnaires and physical examination records in a comprehensive tertiary hospital. The questionnaires included demographic characteristics, work situation and daily lifestyle. Logistic regression and a nomogram were used to develop and validate the prediction model.

**Results:**

A total of 824 female nurses were included in this study. Living situation, smoking history, monthly night shift, daily sleep time, ALT/AST, FBG, TG, HDL-C, UA, BMI, TBil and Ca were independent risk factors for NAFLD occurance. A prediction model for predicting the prevalence of NAFLD among female nurses was developed and verified in this study.

**Conclusion:**

Living situation, smoking history, monthly night shift, daily sleep time, ALT/AST, FBG, TG, UA, BMI and Ca were independent predictors, while HDL-C and Tbil were independent protective indicators of NAFLD occurance. The prediction model and nomogram could be applied to predict the prevalence of NAFLD among female nurses, which could be used in health improvement.

**Trial registration:**

This study was a part of the Chinese Nurse Cohort Study (The National Nurse Health Study, NNHS), which was a ambispective cohort study contained past data and registered at Clinicaltrials.gov (https://clinicaltrials.gov/ct2/show/NCT04572347) and the China Cohort Consortium (http://chinacohort.bjmu.edu.cn/project/102/).

**Supplementary Information:**

The online version contains supplementary material available at 10.1186/s12876-024-03121-1.

## Introduction

Nonalcoholic fatty liver disease (NAFLD) is gradually becoming a huge threat to public health [1]. More than 25% of the population has been diagnosed with NAFLD around the world, and the prevalence increases to 32% in the Middle East and 31% in South America [2]. The epidemic of NAFLD is also severe in China. As the fastest growing country, the number of patients with NAFLD will reach 314.58 million in 2030 [3]. In addition to affecting the structure and function of the liver, NAFLD also has important effects on other organs [4, 5]. Patients with NAFLD are found to have a high risk of developing type 2 diabetes, cardiovascular and cerebrovascular diseases, chronic kidney diseases, and even death from related diseases [4, 5], [6, 7]. Because early NAFLD is preventable and reversible [7], identifying risk factors for NAFLD and providing related intervention are essential.

Previous studies have explored risk factors for NAFLD and found that age, sex, race, metabolic syndrome (MS), unhealthy lifestyle, such as unbalanced diet, sedentary and low-level physical activity and lack of sleep, were tightly associated with the prevalence of NAFLD [8]. Furthermore, several prediction models of NAFLD occurance have been developed, which found that age, ethnicity, sex, exercise, smoking, heart rate, blood pressure, body mass index (BMI), waist circumference, high-density lipoprotein-cholesterol (HDL-C) and bilirubin could independently predict the prevalence of NAFLD [9, 10]. However, most of the current models only focus on laboratory tests while neglecting lifestyle could also influence the prevalence of NAFLD.

As a group with high-intensity and night-shift jobs, nurses have been found to have a high prevalence of NAFLD, especially for nurses working in emergency departments, whose prevalence of NAFLD could increase to 28.3% [11]. Compared to the general population, nurses experience greater work pressure and quite different lifestyles, such as frequent night shifts and extra meals at night, which are high-risk influencing factors of NAFLD [8]. Meanwhile, nurses also experienced more frequent physical activities for constant patient care, which was a protective factor against NAFLD [8]. Moreover, females make up the vast majority of nurses, and 97.7% of nurses are female in China [12]. For complex working characteristics, it is necessary to develop a special prediction model to predict the prevalence of NAFLD in female nurses.

Therefore, the purposes of this study were to identify risk factors for NAFLD from demographic characteristics, work situation, daily lifestyle and physical examination records to develop a prediction model to predict the prevalence of NAFLD in female nurses to guide them prevent and treat NAFLD accurately.

## Methods

### Study design

This study was a part of the Chinese Nurse Cohort Study (The National Nurse Health Study, NNHS), and registration information for this cohort was included in the protocol for the study [13]. The NNHS has been approved by the Medical Research Ethics Committee of Peking University Third Hospital (IRB00006761-M2020306). The Transparent Reporting of a Multivariable Prediction Model for Individual Prognosis or Diagnosis (TRIPOD) statement was applied to standardize the study procedure [14].

### Participants

Female nurses registered and licenced practiced in a comprehensive tertiary hospital were recruited, and data were collected from ^1^ June to ^1^ September 2021 as the first-year follow data of the NNHS. Nurses who were unwilling to participate in research or with missing data were excluded. Informed consent was obtained from all the participants.

### Outcomes

#### Main outcomes

The prevalence of NAFLD was the main outcome of our study. According to the Guidelines for prevention and treatment of nonalcoholic fatty liver disease in China [4], the diagnostic criteria of NAFLD in this study: [1] there was no history of drinking alcohol or the alcohol equivalent amount was less than 70 g/week [2]; diseases that can lead to fatty liver such as viral hepatitis, drug-induced liver disease, and autoimmune liver disease were excluded[3]; imaging of diffuse hepatic steatosis. In this study, the results of abdominal B-ultrasound were used as imaging evidence.

#### Predictors and measurement

The predictors are mainly the following five aspects: demographic characteristics, work situation, daily lifestyle and physical examination record.

Demographic characteristics included age, nationality, education year, living situation, marital status, constipation, laxative drug use, oral contraceptive use, smoking history and drinking history.

Work situation included department, service year, human resources, power of work, monthly income, monthly night shift and work pressure.

Daily lifestyle included exercise, frequency of midnight eating, daily sleep time and sleep disorder.

And Physical examination records included height, weight, body fat weight, skeletal muscle weight, blood pressure (BP), heart rate (HR), alanine aminotransferase (ALT), aspartate aminotransferase (AST), ALT/AST, fasting blood glucose (FBG), total cholesterol (TC), triglyceride (TG), HDL-C, low-density lipoprotein-cholesterol (LDL-C), uric acid (UA), creatinine (Cr), urea nitrogen (Urea), homocysteine (HCY), total bilirubin (TBil) and blood calcium (Ca).

The measurement of those indicators were presented on Table S1.

### Statistical analysis

IBM Statistical Version 23.0 (SPSS, Chicago, USA) and R software Version 4.2.1 (R Foundation, Vienna, Austria) containing Packages “rms”, “pROC”, “rmda”, “nricens” and “ggplot2” were used for data description and statistical analysis. The Q test was used for outlier testing. Classified variables were described as percentage (%), and continuous variables were expressed as mean and standard deviation or median (quartiles). For classified variables, using the chi-square test was used for analysing, and continuous variables were analysed by independent sample t tests or ANOVA. According to the results of univariate analysis in the development set and validation set, factors significantly associated with NAFLD occurance were included in binary logistic regression analysis in the development set (*P* < 0.10). Factors that were significantly associated with NAFLD occurance by binary logistic regression analysis were included in the prediction model (*p* < 0.05). Odds ratios (ORs) and 95% confidence intervals (CIs) were calculated.

A nomogram was drawn based on the results of the binary regression analysis and the prediction model was developed. The discrimination ability of the model was evaluated by the Harrell consistency index (C-index) and receiver operating characteristic (ROC) curve. The calibration of the prediction model was evaluated by calibration curves. The clinical utility of the prediction model was evaluated by decision curve analysis (DCA). The C-index, AUROC and calibration curves were analysed by1000 bootstrap resamples.

## Results

### Participants

**Fig. 1:**
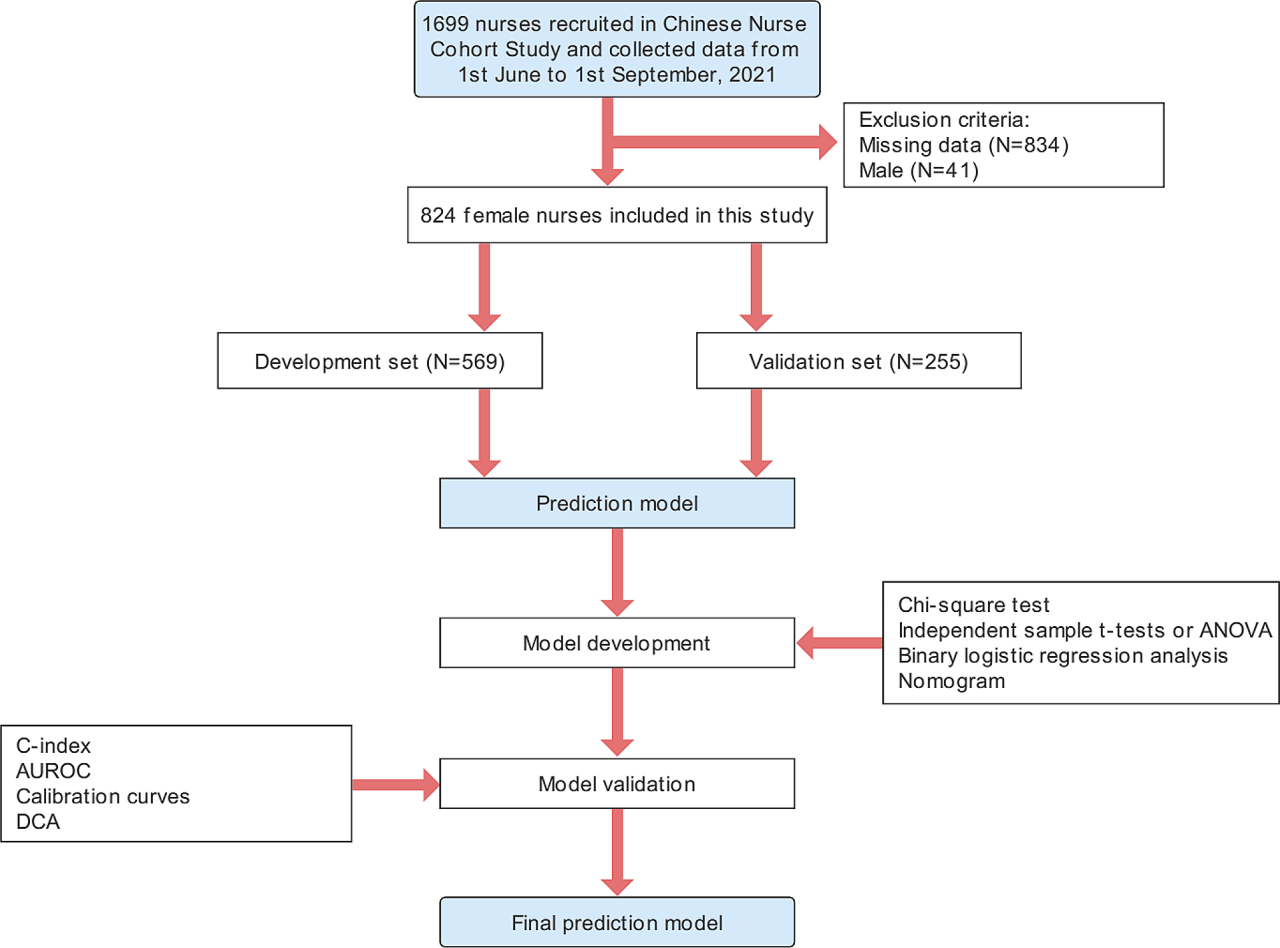
Flow chart of this study

A total of 824 female nurses were included in our study, while 569 were included in the development criteria and 255 were included in the validation set. The follow chart was shown in Fig. 1. The mean age of the total participants was 32.62 ± 7.07 years, and the service year was 11 [7, 17] years, in which the prevalence of NAFLD was 15.5% (128/824). In the development set, the mean age was 32.66 ± 6.84 years, and the prevalence of NAFLD was 15.8% (90/565). In the validation set, the mean age was 32.54 ± 7.56 years, and the prevalence of NAFLD was 14.9% (38/255). There was no significant difference in characteristics between the development set and validation set (Table S2). The characteristics of participants with NAFLD and non-NAFLD are presented in Table 1.

**Table 1:** Characteristics of NAFLD and non-NAFLD patients in the development set and validation set

					Development Set			Validation Set		
					NAFLD	Non-NAFLD	*P*	NAFLD	Non NAFLD	*P*
**Demographic characteristic, N (%)**										
Age			< 30		20 (9.2)	197 (90.8)	< 0.001	7 (6.8)	96 (93.2)	0.001
			30–39		40 (14.7)	232 (85.3)		18 (16.2)	93 (83.8)	
			40–49		23 (34.3)	44 (65.7)		8 (26.7)	22 (73.3)	
			≥ 50		7 (53.8)	6 (46.2)		5 (45.5)	6 (54.5)	
Living situation			Solitude		8 (8.6)	85 (91.4)	0.020	2 (5.3)	36 (94.7)	0.013
			With spouse		61 (17.7)	284 (82.3)		31 (20.8)	118 (79.2)	
			With parents		20 (20.2)	79 (79.8)		5 (9.6)	47 (90.4)	
			With relatives or friends		1 (3.1)	31 (96.9)		0 (0.0)	16 (100.0)	
Marital status			Single		21 (10.6)	178 (89.4)	0.012	3 (3.1)	93 (96.9)	< 0.001
			Married		69 (18.6)	301 (81.4)		35 (22.0)	124 (78.0)	
Smoke history			Yes		84 (15.2)	467 (84.8)	0.018	36 (14.9)	206 (85.1)	0.933
			No		3 (60.0)	2 (40.0)		1 (20.0)	4 (80.0)	
			Passive smoking		3 (23.1)	10 (76.9)		1 (12.5)	7 (87.5)	
**Work situation, N (%)**										
Work department			Inpatient ward		75 (18.3)	335 (81.7)	0.019	31 (15.8)	165 (84.2)	0.847
			Outpatients		5 (7.4)	63 (92.6)		3 (15.0)	17 (85.0)	
			Operating room		8 (17.4)	38 (82.6)		2 (9.5)	19 (90.5)	
			Emergency and critical care unit		2 (4.4)	43 (95.6)		2 (11.1)	16 (88.9)	
Service year			< 5		4 (6.0)	63 (94.0)	< 0.001	2 (4.3)	45 (95.7)	< 0.001
			5–9		19 (11.0)	153 (89.0)		3 (4.9)	58 (95.1)	
			10–19		38 (16.1)	198 (83.9)		16 (16.8)	79 (83.2)	
			≥ 20		29 (30.9)	65 (69.1)		17 (32.7)	35 (67.3)	
Human resource			Contract system		36 (12.2)	258 (87.8)	0.016	17 (12.3)	121 (87.7)	0.208
			Establishment		54 (19.6)	221 (80.4)		21 (17.9)	96 (82.1)	
Monthly night shift			≤ 5		43 (13.5)	275 (86.5)	0.091	18 (13.5)	115 (86.5)	0.522
			> 5		47 (18.7)	204 (81.3)		20 (16.4)	102 (83.6)	
ERI					1.16 ± 0.49	1.05 ± 0.31	0.007	1.17 ± 0.40	1.06 ± 0.33	0.059
**Daily lifestyle, N (%) / Mean ± SD**										
Exercise habit	Yes				72 (18.0)	327 (82.0)	0.026	31 (17.7)	144 (82.3)	0.062
	No				18 (10.6)	152 (89.4)		7 (8.8)	73 (91.3)	
Sleep disorder					7.27 ± 2.59	7.63 ± 2.48	0.208	8.92 ± 2.53	7.94 ± 2.41	0.022
Daily sleep time				< 5 h	8 (36.4)	14 (63.6)	0.024	1 (25.0)	3 (75.0)	0.128
				5–6 h	42 (16.7)	210 (83.3)		26 (20.0)	104 (80.0)	
				7–8 h	36 (12.9)	244 (87.1)		10 (8.7)	105 (91.3)	
				9–10 h	3 (23.1)	10 (76.9)		1 (25.0)	3 (75.0)	
				≥ 11 h	1 (50.0)	1 (50.0)		0 (0.0)	2 (100.0)	
**Laboratory tests, Mean ± SD**										
BMI (kg/m^2^)					26.50 ± 3.81	21.92 ± 2.90	< 0.001	26.18 ± 3.11	21.88 ± 2.54	< 0.001
HR (times/min)					85.78 ± 9.85	83.17 ± 11.04	0.037	81.84 ± 11.48	83.95 ± 10.15	0.208
ALT/AST		≤ 1.33			66 (12.5)	461 (87.5)	< 0.001	28 (11.7)	211 (88.3)	< 0.001
		> 1.33			24 (57.1)	18 (42.9)		10 (62.5)	6 (37.5)	
SBP (mmHg)					125.18 ± 11.87	118.35 ± 10.08	< 0.001	123.55 ± 12.72	118.73 ± 10.94	0.001
DBP (mmHg)					77.77 ± 9.13	72.54 ± 8.19	< 0.001	77.02 ± 8.94	72.95 ± 8.17	0.001
FBG (mmol/L)					5.76 ± 1.70	4.87 ± 0.42	< 0.001	5.19 ± 0.67	4.95 ± 0.68	0.032
TC (mmol/L)					5.02 ± 0.87	4.54 ± 0.85	< 0.001	4.99 ± 0.94	4.48 ± 0.76	0.001
TG (mmol/L)					1.94 ± 2.35	0.89 ± 0.46	< 0.001	1.64 ± 1.01	0.92 ± 0.77	< 0.001
HDL-C (mmol/L)					1.22 ± 0.26	1.52 ± 0.30	< 0.001	1.22 ± 0.26	1.50 ± 0.26	< 0.001
LDL-C (mmol/L)					3.40 ± 0.74	2.85 ± 0.71	< 0.001	3.41 ± 0.81	2.77 ± 0.67	< 0.001
UA (µmol/L)					309.67 ± 57.70	255.50 ± 53.60	< 0.001	302.89 ± 49.77	255.88 ± 44.71	< 0.001
Cr (µmol/L)					63.68 ± 11.17	63.79 ± 7.67	0.930	62.24 ± 7.38	65.05 ± 8.59	0.429
Urea (mmol/L)					4.43 ± 1.22	4.24 ± 1.02	0.115	4.55 ± 0.92	4.28 ± 1.11	0.119
HCY (µmol/L)					10.06 ± 3.42	9.38 ± 2.75	0.361	10.39 ± 4.26	12.92 ± 8.41	0.254
TBil (µmol/L)					12.23 ± 3.97	15.68 ± 5.84	< 0.001	13.25 ± 5.62	14.52 ± 4.95	0.725
Ca (mmol/L)					2.37 ± 0.09	2.34 ± 0.10	0.014	2.34 ± 0.08	2.34 ± 0.09	0.116
Body fat (kg)					25.15 ± 7.32	18.07 ± 5.41	< 0.001	25.11 ± 5.47	18.07 ± 4.58	< 0.001
Skeletal muscle (kg)					23.61 ± 2.32	21.69 ± 3.32	< 0.001	24.24 ± 2.67	21.76 ± 2.26	< 0.001

BMI, body mass index, HR, heart rate, ALT, alanine aminotransferase, AST, aspartate aminotransferase, SBP, systolic blood pressure, DBP, diastolic blood pressure, FBG, fasting blood glucose, TC, total cholesterol, TG, triglyceride, HDL-C, high-density lipoprotein-cholesterol, LDL-C, low-density lipoprotein-cholesterol, UA, uric acid, Cr, creatinine, urea, urea nitrogen, HCY, homocysteine, TBil, total bilirubin, Ca, blood calcium

### Model development

Based on the results of univariate analysis, 26 indicators were recognized as risk factors for NAFLD indicators in the development or validation set, in which 4 were demographic characteristics, 5 were work situation, 2 were daily lifestyle and 15 were laboratory tests (*p* < 0.10) (Table 1). The detailed parameters of the characteristics in univariate analysis are presented in Table 1.

**Fig. 2:**
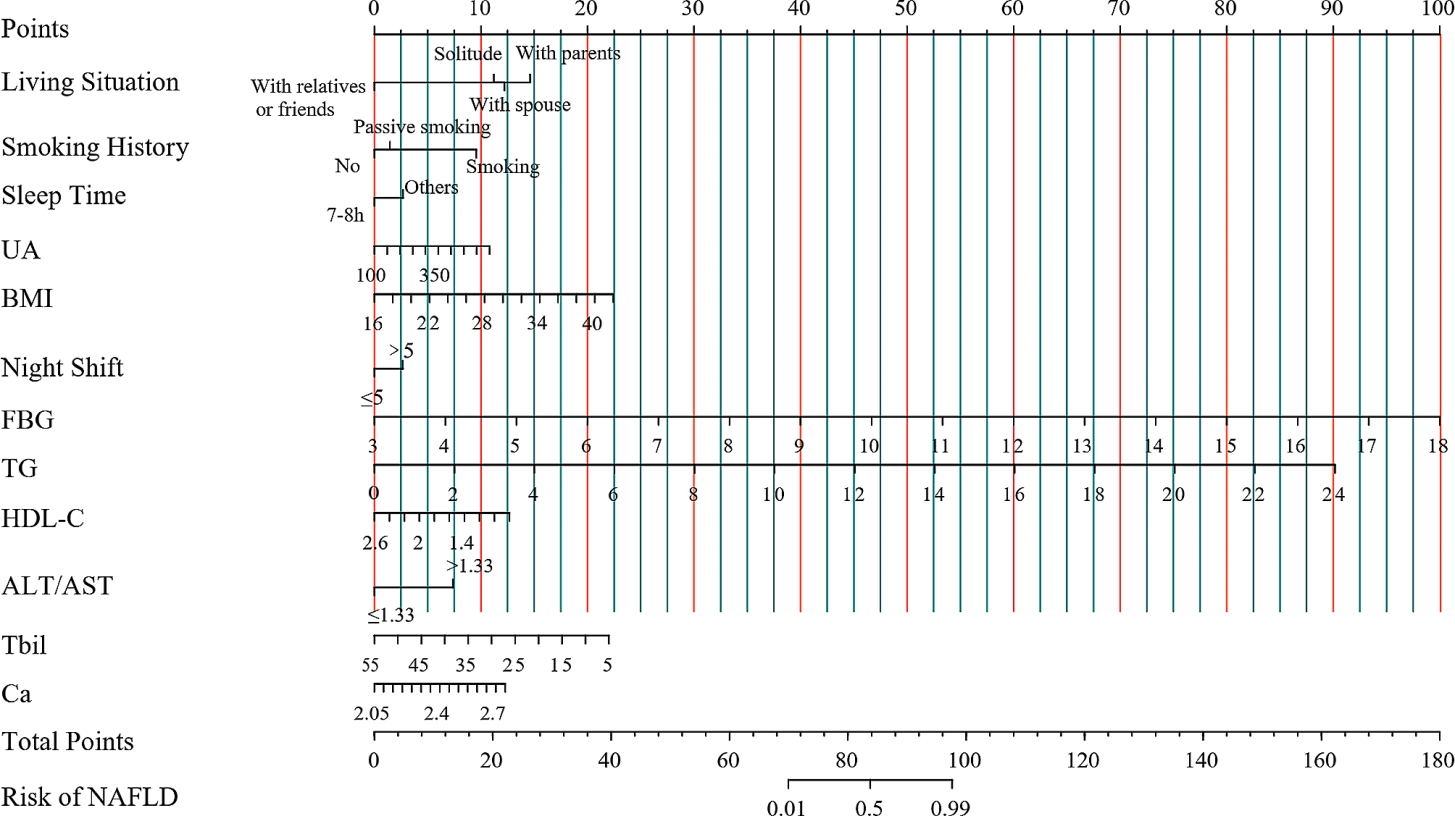
Developed nomogram for predicting the prevalance of NAFLD among nurse

As a result of binary logistic regression analysis, 10 indicators were recognized as risk factors for NAFLD development (*p* < 0.05) (Table 2). The results of binary logistic regression analysis showed that living situation, smoking history, monthly night shift, daily sleep time, ALT/AST, FBG, TG, HDL-C, UA, BMI, TBil and Ca were independent risk factors for NAFLD prevalence. Then, the NAFLD risk nomogram was built based on the 10 independent predictors described above (Fig. 2).

**Table 2:** The results of binary logistic regression analysis

	B	SE	Wald	df	*P* value	OR	95% confidence interval	
							Lower	Upper
Age (Compared with < 30)			2.038	3	0.564			
Age (30–39 years)	0.215	0.859	0.063	1	0.802	1.240	0.230	6.685
Age (40–49 years)	2.200	1.872	1.381	1	0.240	9.029	0.230	354.161
Age (≥ 50 years)	2.914	2.090	1.944	1	0.163	18.439	0.307	1109.000
Living situation (Compared with Living with relatives or friends)			11.092	3	0.011			
Living situation ( Solitude )	4.620	2.072	4.973	1	0.026	101.490	1.750	5886.853
Living situation (With spouses)	5.579	2.130	6.862	1	0.009	264.693	4.074	17195.810
Living situation (With parents)	6.368	2.068	9.477	1	0.002	582.702	10.110	33585.722
Marital status (Compared with single)	-1.373	0.860	2.545	1	0.111	0.253	0.047	1.369
Smoking history (Compared with no)			3.983	2	0.136			
Smoking history (Yes)	3.619	1.813	3.983	1	0.046	37.300	1.067	1303.894
Smoking history (Passive smoking)	− 0.007	1.111	0.000	1	0.995	0.993	0.113	8.763
Work department (Compared with Inpatient ward)			4.200	3	0.241			
Work department (Outpatients ward)	-1.861	0.989	3.542	1	0.060	0.156	0.022	1.080
Work department (Operating room)	− 0.949	1.108	0.733	1	0.392	0.387	0.044	3.400
Work department (Emergency and critical care unit)	0.304	0.947	0.103	1	0.748	1.355	0.212	8.673
Service years (Compared with < 5 years)			4.177	3	0.243			
Service years (5–9 years)	2.194	1.177	3.473	1	0.062	8.975	0.893	90.216
Service years (10–19 years)	2.228	1.378	2.614	1	0.106	9.277	0.623	138.104
Service years (≥ 20 years)	0.711	2.078	0.117	1	0.732	2.037	0.035	119.630
Human resource (Compared with contract system)	− 0.461	0.578	0.635	1	0.426	0.631	0.203	1.959
Monthly night shift (Compared with ≤ 5)	1.256	0.515	5.954	1	0.015	3.512	1.280	9.633
ERI (Compared with No)	− 0.566	0.475	1.420	1	0.233	0.568	0.224	1.440
Exercise habit (Compared with no)	0.198	0.511	0.150	1	0.699	1.219	0.448	3.317
Daily sleep time (Compared with 7–8 h)	-1.046	0.528	3.920	1	0.048	0.351	0.125	0.990
BMI	0.488	0.136	12.806	1	0.000	1.628	1.247	2.127
HR	0.034	0.025	1.941	1	0.164	1.035	0.986	1.086
ALT/AST	2.612	0.796	10.776	1	0.001	13.622	2.864	64.784
SBP	− 0.029	0.031	0.843	1	0.359	0.972	0.913	1.033
DBP	0.000	0.039	0.000	1	0.993	1.000	0.927	1.079
FBG	2.330	0.550	17.917	1	0.000	10.280	3.495	30.239
TC	− 0.053	0.747	0.005	1	0.944	0.949	0.219	4.104
TG	1.212	0.400	9.205	1	0.002	3.361	1.536	7.354
LDL-C	0.761	0.796	0.915	1	0.339	2.141	0.450	10.195
HDL-C	-2.849	1.263	5.088	1	0.024	0.058	0.005	0.688
UA	0.008	0.004	4.245	1	0.039	1.008	1.000	1.016
TBil	− 0.199	0.067	8.808	1	0.003	0.819	0.718	0.935
Ca	6.093	2.574	5.602	1	0.018	442.799	2.850	68794.437
Body fat	− 0.087	0.070	1.545	1	0.214	0.917	0.800	1.051
Skeletal muscle	0.021	0.134	0.024	1	0.876	1.021	0.785	1.328
Constant	-43.962	9.091	23.384	1	0.000	0.000		

### Model validation

The C-index of the nomogram in the development set and validation set were 0.97 and 0.93, respectively, which indicated that the nomogram had pretty discrimination and prediction abilities. The AUROC of the nomogram in the development set was 0.97 (95% CI, 0.96-0.99), while the sensitivity was 0.93 and the specificity was 0.93. The AUROC of the nomogram in the validation set was 0.98 (95% CI, 0.97-0.98), while the sensitivity and specificity were 0.97 and 0.90, respectively (Figure S1). Therefore, the nomogram performed well.

The calibration curves of the development set and validation set are presented in Figure S2 and indicated that the nomogram had good agreement between the predicted probabilities and the actual observed probabilities. DCA showed that the application of the nomogram in female nurses to predict the risk of NAFLD was more effective than the intervention-for-all-patients scheme (Figure S3).

## Discussion

To our knowledge, this may be the first study to investigate the prevalance and influencing factors of NAFLD and develop a prediction model in female nurses. Based on the baseline NNHS data, we developed and validated a prediction model for predicting the prevalence of NAFLD in female nurses. Meanwhile, we developed an intuitive nomogram to visualize predictive models for clinical use. The model displayed excellent discrimination and clinical value. Predictors in the model included BMI, FBG, TG, HDL, AST, ALT, Tbil, UA and monthly night shift, which contained some risk factors consistent with previous studies NAFLD [9, 10]. and one factor reflecting the professional characteristics of nurses.

In this study, the prevalence of NAFLD was 15.5%, which was slightly lower than previous studies conducted in healthy participants or patients with T2DM [15]. Although previous studies found that the prevalence of NAFLD was higher among nurses [11], we failed to find similar results. This may be the result of different department distributions, while only 7.65% of female nurses included in this study were working in emergency and critical care units. Furthermore, only females were recruited in this study, but males had been found to have a higher risk of NAFLD (OR 1.779, 95% CI 1.676–1.888) [9]. The participants included in this study were much younger than those in previous studies, while older age was an independent predictor of NAFLD occurance [9, 16]. These may also be the reasons for the results.

Many previous studies have proven that obesity is an important risk factor for NAFLD [17, 18]. A study in China showed that the risk of NAFLD increases with BMI, even in nonobese individuals [10]. Similar conclusions also appeared in our research. Meanwhile, T2DM has also been found to be a prodictor for NAFLD [17]. In our study, FBG was still the strongest predictor of the prevalence of NAFLD, regardless of whether female nurses had diabetes.

Increased TGs and decreased HDL-C concentrations always appeared in NAFLD [19], as did our study. As the predominant form of fat accumulation in the liver, increased TG has been found to be strongly associated with NAFLD [20]. HDL is a substance that transports triglycerides in the liver, so a high concentration of HDL is a protective factor for NAFLD, while previous studies also showed that TG/HDL-C may be a good predictor of NAFLD [21].

AST and ALT can be increased without accompanying symptoms. ALT is most closely related to liver fat accumulation, even within the normal reference range [22], and is often used as a surrogate marker for NAFLD in epidemiological studies [23]. Recent studies have shown that the ALT/AST ratio may be more sensitive and specific as a marker of NAFLD than ALT and AST alone, especially for patients with normal ALT and AST [22]. Similarly, we found that higher ALT/AST were related to an increased prevalence of NAFLD, which was consistent with previous studies.

Furthermore, we also found that bilirubin was a protective factor against the occurance of NAFLD. As the end product of heme metabolism, the beneficial properties of bilirubin have attracted increasing attention, such as antioxidant and anti-inflammatory effects [24]. Meanwhile, oxidative stress and the inflammatory response have been proven to be important contributors to the pathogenesis of NAFLD [4]. Therefore, higher bilirubin may be a protective facto of NAFLD via antioxidant and anti-inflammatory effects. Meanwhile, Higher bilirubin was associated with lower incidence of abdominal obesity and metabolic syndrome, while was abdominal obesity and metabolic syndrome were risk factors of NAFLD [25, 26]. These may be the reason that bilirubin was a protective factor against the occurance of NAFLD.

In addition, we also found that the increase in serum calcium was related to the occurrence of NAFLD, which might be a dose‒response effect. A study of nonalcoholic fatty liver disease in South Korea reached a similar conclusion [27]. Meanwhile, previous studies have also confirmed that serum calcium has a significant correlation with insulin resistance, abnormal glucose metabolism and abnormal lipid metabolism [28]. However, the conclusions of current studies on the relationship between serum calcium and NAFLD are not consistent, and more studies are needed to verify this hypothesis.

The most important finding of our study was that we indicated lifestyle could also influence the prevalence of NAFLD, such as living situation, smoking history and sleep time. Previous studies found that higher ultra-processed food consumption was associated with eating with family members, and eating with friends” was associated with lower ultra-processed food consumption [29]. That may be the reason that living with spouse and parents were associated with higher risk of NAFLD. As for smoking history, compared with no smoking or passive smoking, we found that smoking particiants had much higher risks of NAFLD, which was consistent with previous studies [30, 31]. Although no studies paid attention on the relation between sleep time and NAFLD, trouble sleeping was positively associated with NAFLD [32]. Meanwhile, 7-8 h sleep was also the nadir for associations with all-cause, cardiovascular disease, and other-cause mortality [33].

Meanwhile, we also found night shift was associated with the occurance of NAFLD in female nurses. Previous studies have shown that exposure to light at night may lead to insufficient melatonin secretion and disorders of liver metabolism [34], while Irregular-shift work have been fould that are associated with pathological liver fat accumulation [35]. Meanwhile, prolonged night work has been found that could increase nurses’ risk of dyslipidemia and abnormal liver function [36]. And circadian misalignment may have an underlying pathogenic role. This may be the reason why a more frequent night shift was associated with the prevalence of NAFLD.

Although not included in the model, several indicators of work situation, such as human resources, work department and service years, were also found to be associated with the prevalence of NAFLD in univariate analysis. Meanwhile, exercise habits and weekly family time could also influence the prevalence of NAFLD in univariate analysis. Therefore, it is important to develop a more appropriate shift system to control the monthly shift number and increase family and sleep time, which are challenges for nurse managers and policy makers.

### Strengths and limitations

This study is the first to focus on the predictors of nonalcoholic fatty liver disease in female nurses. Meanwhile, our study is also the first to add nurses’ work characteristics, daily lifestyle and social psychological indicators to the prediction model of NAFLD occurance, which could provide recommendations for the prevention and treatment of NAFLD among nurses and other groups with similar work characteristics. This study also has several limitations. First, the data of this study were obtained from baseline data of NNSH that were collected at one hospital, which may lead to limited representation. In the future, the follow-up data of the NNHS could be used to verify the prediction model, and multicenter studies are also needed. In addition, NAFLD was diagnosed using ultrasonography in this study, which was greatly dependent on the proficiency of doctors. Although the doctors included in this study were experienced, there still existed a possibility of diagnostic bias.

## Conclusion

A prediction model for predicting the prevalence of NAFLD among female nurses was developed and verified in this study. Living situation, smoking history, monthly night shift, daily sleep time, ALT/AST, FBG, TG, UA, BMI and Ca were independent predictors, while HDL-C and Tbil were independent protective indicators of NAFLD occurance. The model displayed excellent discrimination and clinical value, which has clinical significance for identifying the risk of NAFLD and can provide some suggestions for the prevention and treatment of NAFLD.

### Electronic supplementary material

Below is the link to the electronic supplementary material.

**Supplementary material: Table S1** Predictors and Measurements in This Study. **Table S2** Characteristics of the Development Cohort and Validation Set. **Figure S1** ROC curves of the nomogram in the development set and validation set. **Figure S2** Calibration curves of the nomogram. **Figure S3** Decision curves of the nomogram

## Data Availability

The datasets used and/or analysed during the current study are available from the corresponding author on reasonable request.
