# Supplementary material for: Development of a prediction model for predicting the prevalence of nonalcoholic fatty liver disease in Chinese nurses: the first-year follow data of a web-based ambispective cohort study
Source: BMC Gastroenterol. 2024 Feb 14;24:72. doi: 10.1186/s12876-024-03121-1 (PMC10868006; doi:10.1186/s12876-024-03121-1)

Table S1 Predictors and Measurements in This Study

| Predictors | Measurements |
| --- | --- |
| **Demographic characteristics** | |
| Age, nationality, education year, living situation, marital status, constipation, laxative drug use, oral contraceptive use, smoking history and drinking history. | |
| **Work situation** | |
| Department, service year, human resources, power of work, monthly income, monthly night shift | |
| Work pressure | Seven items from the effort-reward imbalance (ERI) scale, which is a 4-point Likert scale including two dimensions: effort and reward. The effort return ratio calculated by the average score of the effort dimension divided by the average score of the reward dimension was used to assess the work pressure, where “<1” meant that there was work pressure and “≥1” meant that there was inexistent work pressure(30). The Cronbach's α coefficient was 0.746 in this study. |
| **Daily lifestyle** | |
| Exercise and Frequency of midnight eating. | |
| Sleep situation-  Daily sleep time | Asking participants how much sleep they average get on every day, and “<5 h”, “5-6 h”, “7-8 h”, “9-10 h”, “≥11 h” could be selected. |
| Sleep situation-  Sleep disorder | Pittsburgh Sleep Quality Index (PSQI), which included 19 self-rated items and 5 other-rated items with seven dimensions. Each dimension was scored from 0 to 3, and the total score ranged from 0 to 21. High scores indicated worse sleep quality, and a total score ≥7 indicated that participants had sleep disorders(31). The Cronbach's α coefficient was 0.768 in this study. |
| **Physical examination records** | |
| Height and weight | Measured by same machine while participants were fasting and slipper, then used to calculate BMI (kg/m^2^) by standard formula. |
| Body fat weight and skeletal muscle weight | Measured by Inbody770. |
| Blood pressure (BP) and Heart rate (HR) | An automatic BP monitor in the seated position. |
| Alanine aminotransferase (ALT),  Aspartate aminotransferase (AST),  ALT/AST,  Fasting blood glucose (FBG),  Total cholesterol (TC),  Triglyceride (TG),  HDL-C,  Low-density lipoprotein-cholesterol (LDL-C),  Uric acid (UA),  Creatinine (Cr),  Urea nitrogen (Urea),  Homocysteine (HCY),  Total bilirubin (TBil),  Blood calcium (Ca) | All of the blood sample were collected by medically trained staff from the medical examination center of the hospital. Participants were asked to fast overnight. After collection, All the blood samples were transported within two hours to the clinical laboratory of the hospital, and standard blood assay were performed during November, 2020 to Match, 2021. ALT/AST was divided to ≤1.33 and >1.33. |

| Table S2 Characteristics of the Development Cohort and Validation Set | | | | | |
| --- | --- | --- | --- | --- | --- |
|  | | | Development Set | Validation Set | *P* |
| NAFLD incidence, N (%) | | | 90(15.8) | 38 (14.9) | .890 |
| **Demographic characteristic, N (%)** | | | | | |
| Age (years) | | <30 | 217 (38.1) ^*^ | 103 (40.4) ^*^ | .332 |
|  |  | 30-39 | 272 (47.8) | 111 (43.5) |  |
|  |  | 40-49 | 67 (11.8) | 30 (11.8) |  |
|  |  | ≥50 | 13 (2.3) | 11 (4.3) |  |
| Nationality | | Han | 526 (92.4) | 238 (93.3) | .757 |
|  |  | Minority | 43 (7.6) | 17 (6.7) |  |
| Education year | | ≤9 | 2 (0.4) | 3 (1.2) | .328 |
|  |  | 12 | 117 (20.6) | 62 (24.3) |  |
|  |  | 13-14 | 444 (78.0) | 187 (73.3) |  |
|  |  | >14 | 6 (1.1) | 3 (1.2) |  |
| Living situation | | Solitude | 93 (16.3) ^*^ | 38 (14.9) ^*^ | .715 |
|  |  | With spouse | 345 (60.6) | 149 (58.4) |  |
|  |  | With parents | 99 (17.4) | 52 (20.4) |  |
|  |  | With relatives or friends | 32 (5.6) | 16 (6.3) |  |
| Marital status | | Single | 199 (35.0) ^*^ | 96 (37.6) ^*^ | .459 |
|  |  | Married | 370 (65.0) | 159 (62.4) |  |
| Constipation | | Yes | 64 (11.2) | 22 (8.6) | .311 |
|  |  | No | 505 (88.8) | 233 (91.4) |  |
| Laxative drug use | | Yes | 122 (21.4) | 53 (20.8) | .831 |
|  |  | No | 447 (78.6) | 202 (79.2) |  |
| Oral contraceptive use | | Yes | 14 (2.5) | 9 (3.5) | .389 |
|  |  | No | 555 (97.5) | 246 (96.5) |  |
| Smoke history | | Yes | 5 (0.9) ^*^ | 5 (2.0) | .346 |
|  |  | No | 551 (96.8) | 242 (94.9) |  |
|  |  | Passive smoking | 13 (2.3) | 8 (3.1) |  |
| Drinking history | | Yes | 42 (7.4) | 26 (10.2) | .175 |
|  |  | No | 527 (92.6) | 229 (89.8) |  |
| **Work situation, N (%)** | | | | | |
| Work department | | Inpatient ward | 410 (72.1) ^*^ | 196 (76.9) | .320 |
|  |  | Outpatients | 68 (12.0) | 20 (7.8) |  |
|  |  | Operating room | 46 (8.1) | 21 (8.2) |  |
|  |  | Emergency and critical care unit | 45 (7.9) | 18 (7.1) |  |
| Service year | | <5 | 67 (11.8) ^*^ | 47 (18.4) ^*^ | .016 |
|  |  | 5-9 | 172 (30.2) | 61 (23.9) |  |
|  |  | 10-19 | 236 (41.5) | 95 (37.3) |  |
|  |  | ≥20 | 94 (16.5) | 52 (20.4) |  |
| Human resource | | Contract system | 294 (51.7) ^*^ | 138 (54.2) | .515 |
|  |  | Establishment | 275 (48.3) | 117 (45.9) |  |
| Power of work | | Brainwork | 55 (9.7) | 24 (9.4) | .895 |
|  |  | Light physical labor | 170 (29.9) | 78 (30.6) |  |
|  |  | Moderate physical labor | 307 (54.0) | 133 (52.2) |  |
|  |  | Heavy physical labor | 37 (6.5) | 20 (7.8) |  |
| Monthly income | | ≤4000 | 1 (0.2) | 0 (0.0) | .318 |
|  |  | 4001-8000 | 63 (11.1) | 35 (13.7) |  |
|  |  | 8001-12000 | 329 (57.8) | 154 (60.4) |  |
|  |  | ≥12000 | 176 (30.9) | 66 (25.9) |  |
| Monthly night shift | | ≤5 | 318 (55.9) ^*^ | 133 (52.2) | .320 |
|  |  | > 5 | 251 (44.1) | 122 (47.8) |  |
| Work pressure | | No | 278 (48.9) | 118 (46.3) ^*^ | .412 |
|  |  | Yes | 291 (51.1) | 137 (53.7) |  |
| **Daily lifestyle, N (%)** | | | | | |
| Exercise habit | | Yes | 170 (29.9) ^*^ | 80 (31.4) ^*^ | .666 |
|  |  | No | 399 (70.1) | 175 (68.6) |  |
| Frequency of  midnight snack | | Never | 285 (50.1) | 119 (46.7) | .620 |
|  |  | Sometimes | 281 (49.4) | 134 (52.5) |  |
|  |  | Always | 3 (0.5) | 2 (0.8) |  |
| Sleep situation | Daily sleep time | <5 h | 22 (3.9) ^*^ | 4 (1.6) | .150 |
|  |  | 5-6 h | 252 (44.3) | 130 (51.0) |  |
|  |  | 7-8 h | 280 (49.2) | 115 (45.1) |  |
|  |  | 9-10 h | 13 (2.3) | 4 (1.6) |  |
|  |  | ≥11 h | 2 (0.4) | 2 (0.8) |  |
|  | Sleep disorder | Yes | 351 (61.7) | 181 (71.0) ^*^ | .010 |
|  |  | No | 218 (38.3) | 74 (29.0) |  |
| **Laboratory tests, Mean±SD/ Median (IQR)** | | | | | |
| BMI (kg/m^2^) | | | 22.65±3.49^*^ | 22.52±3.04^*^ | .621 |
| HR (times/min) | | | 83.58±10.89^*^ | 83.77±10.16 | .767 |
| ALT (U/L) | | | 16.44±12.81 ^*^ | 15.32±9.14^*^ | .312 |
| AST (U/L) | | | 18.94±6.77 ^*^ | 18.77±5.28^*^ | .871 |
| SBP (mmHg) | | | 119.43±10.67^*^ | 119.45±11.32^*^ | .976 |
| DBP (mmHg) | | | 73.37±8.55^*^ | 73.56±8.40^*^ | .771 |
| FBG (mmol/L) | | | 5.00±0.84^*^ | 4.99±0.69^*^ | .763 |
| TC (mmol/L) | | | 4.62±0.87^*^ | 4.56±0.81^*^ | .370 |
| TG (mmol/L) | | | 1.05±1.09 ^*^ | 1.03±0.85 ^*^ | .747 |
| HDL-C (mmol/L) | | | 1.48±0.31^*^ | 1.46±0.28^*^ | .544 |
| LDL-C (mmol/L) | | | 2.94±0.74^*^ | 2.86±0.73^*^ | .188 |
| UA (μmol/L) | | | 264.07±57.71^*^ | 262.89±48.39^*^ | .776 |
| Cr (μmol/L) | | | 63.77±8.31 | 64.62±8.47 | .173 |
| Urea (mmol/L) | | | 4.27±1.05 | 4.32±1.08 | .496 |
| HCY (μmol/L) | | | 9.63±3.01 | 9.10 (7.98,12.18) | .019 |
| TBil (μmol/L) | | | 15.13±5.73 ^*^ | 14.33±5.06 | .055 |
| Ca (mmol/L) | | | 2.34±0.10^*^ | 2.34±0.09 | .653 |
| Body fat (kg) | | | 19.19±6.30^*^ | 19.12±5.34^*^ | .873 |
| Skeletal muscle (kg) | | | 22.00±2.40^*^ | 22.13±2.48^*^ | .459 |

* indicates that this factor was strongly associated with NAFLD prevalence (p<.100); BMI, body mass index, HR, heart rate, ALT, alanine aminotransferase, AST, aspartate aminotransferase, SBP, systolic blood pressure, DBP, diastolic blood pressure, FBG, fasting blood glucose, TC, total cholesterol, TG, triglyceride, HDL-C, high-density lipoprotein-cholesterol, LDL-C, low-density lipoprotein-cholesterol, UA, uric acid, Cr, creatinine, urea, urea nitrogen, HCY, homocysteine, TBil, total bilirubin, Ca, blood calcium.

Figure S1 ROC curves of the nomogram in the development set and validation set


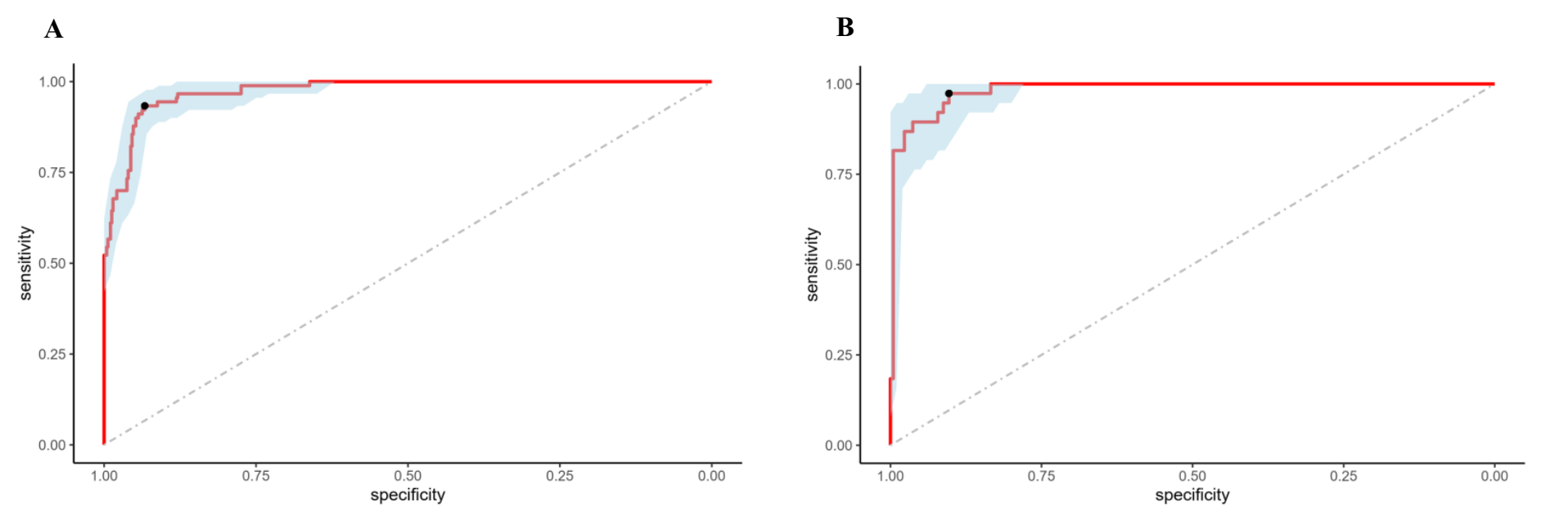


Figure S2 Calibration curves of the nomogram


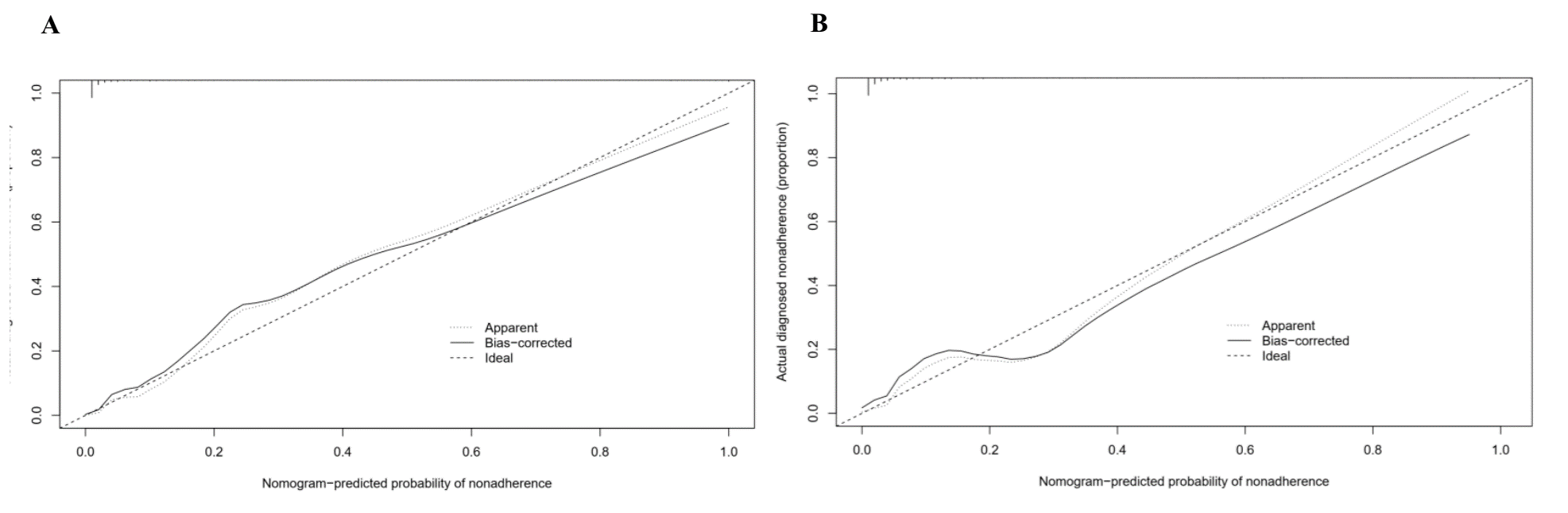


Figure S3 Decision curves of the nomogram


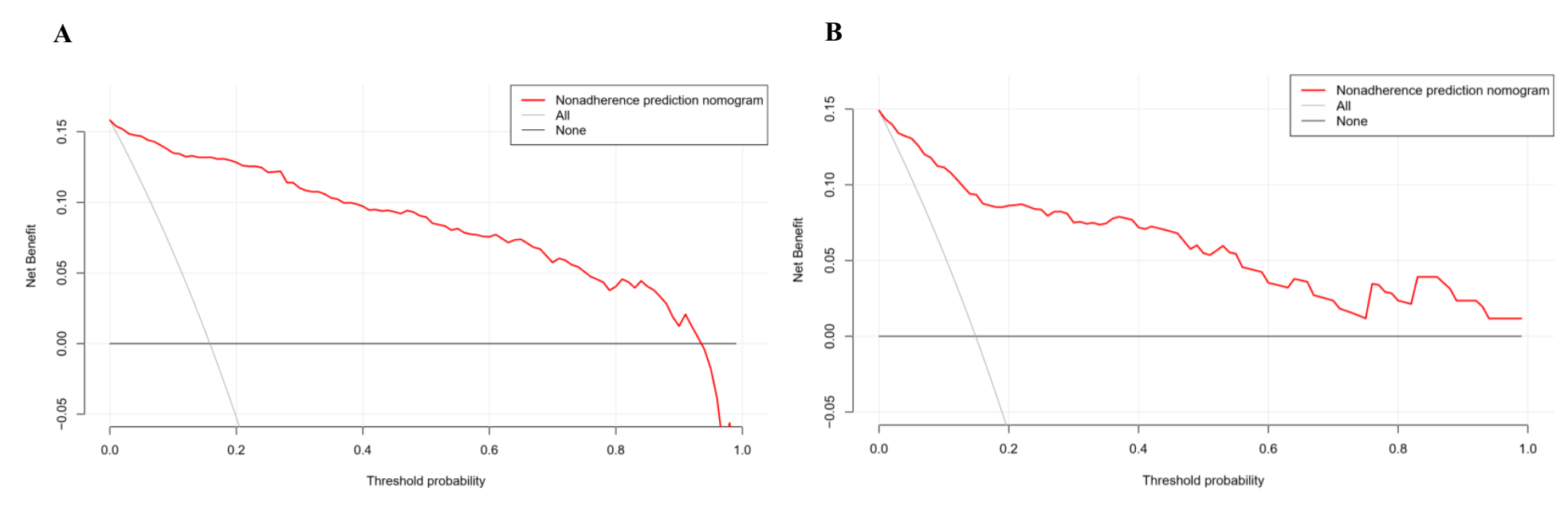

Supplement: Supplementary file 1 — Supplementary material: Table S1 Predictors and Measurements in This Study. Table S2 Characteristics of the Development Cohort and Validation Set. Figure S1 ROC curves of the nomogram in the development set and validation set. Figure S2 Calibration curves of the nomogram. Figure S3 Decision curves of the nomogram [file 12876_2024_3121_MOESM1_ESM.docx]
